# TFEB-lysosome pathway activation is associated with different cell death responses to carbon quantum dots in Kupffer cells and hepatocytes

**DOI:** 10.1186/s12989-022-00474-x

**Published:** 2022-04-28

**Authors:** Yanting Pang, Ying Yao, Mengran Yang, Daming Wu, Ying Ma, Yuanjian Zhang, Ting Zhang

**Affiliations:** 1 Key Laboratory of Environmental Medicine and Engineering, Ministry of Education, School of Public Health, Southeast University, Nanjing, 210009 China; 2 Yangzhou Center for Disease Prevention and Control, Yangzhou, 225200 Jiangsu China; 3 Jiangsu Engineering Laboratory of Smart Carbon-Rich Materials and Devices, Jiangsu Province Hi-Tech Key Laboratory for Bio-Medical Research, School of Chemistry and Chemical Engineering, Southeast University, Nanjing, 211189 China

**Keywords:** Hepatotoxicity, Carbon quantum dots, Lysosomal stress, Autophagic flux, TFEB pathway

## Abstract

**Background:**

Carbon dot has been widely used in biomedical field as a kind of nanomaterial with low toxicity and high biocompatibility. CDs has demonstrated its unique advantages in assisted drug delivery, target diagnosis and targeted therapy with its small size and spontaneous fluorescence. However, the potential biosafety of CDs cannot be evaluated. Therefore, we focused on the study of liver, the target organ involved in CDs metabolism, to evaluate the risk of CDs in vitro.

**Methods and results:**

Liver macrophage KUP5 cells and normal liver cells AML12 cells were incubated in CDs at the same concentration for 24 h to compare the different effects under the same exposure conditions. The study found that both liver cell models showed ATP metabolism disorder, membrane damage, autophagosome formation and lysosome damage, but the difference was that, KUP5 cells exhibited more serious damage than AML12 cells, suggesting that immunogenic cell type is particularly sensitive to CDs. The underlying mechanism of CDs-induced death of the two hepatocyte types were also assessed. In KUP5 cells, death was caused by inhibition of autophagic flux caused by autophagosome accumulation, this process that was reversed when autophagosome accumulation was prevented by 3-MA. AML12 cells had no such response, suggesting that the accumulation of autophagosomes caused by CDs may be specific to macrophages.

**Conclusion:**

Activation of the TFEB-lysosome pathway is important in regulating autophagy and apoptosis. The dual regulation of ERK and mTOR phosphorylation upstream of TFEB influences the death outcome of AML12 cells. These findings provide a new understanding of how CDs impact different liver cells and contribute to a more complete toxicological safety evaluation of CDs.

## Background

Carbon dots (CDs) have several important properties, including chemical inertness, biocompatibility, long fluorescence life, and photostability [1]. Compared with cadmium-containing quantum dots, CDs are being considered as an excellent alternative to metal-based luminescent materials because they are biodegradable and environmentally friendly, inexpensive, and easy to synthesize [2, 3]. CDs have application potential for biomedicine, specifically in drug delivery, photocatalysis, biosensing and photodynamic therapy [4]. However, the application of carbon nanoparticles is still relatively limited due to insufficient knowledge about their biological reactivity. For example, studies have found that pristine carbon nanotubes or C_60_-fullerenes and other carbon nanomaterials may cause toxicity while exerting biological reactivity due to their small size. In addition, carbon-based materials may leave a residue during cell imaging and drug delivery, potentially accumulating in target organs. These toxicological risks cannot be ignored. Thus, it is important to study the damage carbon nanoparticles may cause to different target organs or cell types and to evaluate both their effectiveness and safety before using them in living organisms.

Biological distribution can be used as an important research method to analyze pharmacokinetics. Studies have found that graphitic carbon nitrides (g-C3N4) injected intravenously into the body mainly accumulated in the lungs 2 h after exposure, and when the time extended to 24 h, the liver became the main uptake site (71.37%) [5]. Moreover, the liver contains the mononuclear phagocyte system (MPS) with rich population of macrophages (Kupffer cells) that take up nanoparticles. There are still some studies have found that pristine or functionalized CDs are mainly accumulated in the liver [6] and lungs [7] and may even cross the blood–brain barrier into the brain [8–10]. CDs accumulation in the liver is as high as 52% after one week of exposure, and the liver excretes CDs primarily through the intestine [11, 12]. It follows that liver is the main member involved in the removal of xenobiotics. The liver is an important metabolic detoxification organ composed of 15% macrophages and 60–80% parenchyma cells [13, 14]. Thus, the regulatory role of these cell types in the liver is important for metabolic clearance of exogenous toxins. Hepatic macrophages known as Kupffer cells (KUP5 cells or KCs), play a key role in immune defense, participating in phagocytosis and submitting immune response signals in the early stages of chemical exposure. KUP5 cells are an important component of the monocyte-macrophage system, serving as the first line of defense against foreign substances, and belong to the macrophage type inherent in liver tissue [15]. In contrast, liver parenchymal cells constitute the main body of the liver and play a critical part in detoxification, metabolism, and endocrine function [13]. Studies show that CDs can affect cell division and proliferation by interfering with the expression of DNA damage repair and cell cycle regulation genes [16]. These particles can stably exist in the liver for 28 days and can cause inflammatory responses by inducing oxidative stress in liver cells [17]. In addition, Cu, N-doped CDs can induce endoplasmic reticulum stress and aggravate cell apoptosis, and cause contraction deformation and surface cavity formation [18] that ultimately destroys a cell’s protective barrier. However, recent toxicological safety evaluations of CDs are ambiguous. For example, while Shoval et al. [19] found that CDs did not have a toxic effect in mice and could be used as an ideal material for antibacterial treatment [20], Zhang et al. [21] found that N-doped CDs induce Hepa1-6 cells to produce protective autophagy in response to damage by oxygen free radicals. Collectively, while CDs appear to have low toxicity and strong biocompatibility, there is not enough evidence to conclude that CDs exposure is safe for normal liver tissue. Moreover, most current studies are based on the single-cell model, and existing studies mainly focus on the description of the physical and chemical properties of CDs. The impact of initial exposure or a sublethal level of CDs on different liver cell types remains unknown. Thus, it is critical to evaluate the impact of different levels of CDs exposure to the two main liver cell types.

As a zero-dimensional material, CDs are superior to common one-dimensional, two-dimensional and three-dimensional carbon nanomaterials at crossing biological barriers, and have higher exposure risk due to their small size. A recent study found that carbon-based nanomaterials can induce chronic inflammation, interfering with endocrine functioning and xenobiotic metabolism [22], and causing fibrosis and carcinogenesis. The toxicity of carbon-based materials is mainly attributed to the generation of reactive oxygen species (ROS) triggered by carbon-based materials [23], resulting in DNA damage [24], mitochondrial dysfunction, inflammation, and apoptosis [25]. Cell damage eventually leads to different types of cell death, of which apoptosis is the primary mode and considered an important target of anti-tumor therapy [26]. However, when a large number of cells undergo apoptosis this can negatively impact normal functioning. In addition, autophagy is a regulatory process that plays an important role in the survival, differentiation, and development of cells, allowing for damaged, harmful, or obsolete cellular components to be degraded and recycled [27]. Recent studies have found that carbon nanomaterials can activate autophagy signaling pathways through different processes [28], such as endoplasmic reticulum stress, ROS accumulation, polyubiquitinated proteins, and mitochondrial dysfunction [29]. Interestingly, oxidative stress caused by ROS or mitochondrial damage can also lead to apoptosis [30], suggesting that autophagy and apoptosis may be interrelated. In addition, it has been found that the accumulation of autophagosome can promote the occurrence of apoptosis [27, 31, 32], and autophagy and apoptosis can be cross-inhibited each other [33]. However, relatively few studies have assessed the hepatotoxicity caused by CDs, so it is not known how CDs exposure causes the death of different liver cell types. Thus, the current study compared the autophagy process of KUP5 cells and AML12 cells in response to CDs exposure and further characterized the relationship between autophagy and apoptosis in these two liver cell types.

Lysosomes are intracellular purifiers that participate in the circulation of intracellular organic matter and the removal of damaged organelles [34]. Lysosomes have an acidic interior and contain a high number of hydrolases [35], so exogenous substances or endogenous metabolites are most often transferred to lysosomes for dissolution, metabolism, or temporary storage. In addition, synthetic CDs have excellent biocompatibility and water solubility, so specific binding between synthetic CDs and lysosomes has been extensively studied in targeted imaging and intracellular localization experiments [36, 37]. However, studies have found that CDs ingested by liver cells can aggregate in lysosomes and induce lysosomal stress [21, 38]. CDs ingestion by lysosomes correlates with intracellular ROS production and autophagy, and can serve as an important trigger of autophagy [21]. Autophagy is regarded as the sensitive markers of cellular stress response [39], and autophagic flux is an orderly process start from lysosomal stress induced autophagosome formation [40] to autophagy-lysosome degradation, any link disorders are likely to lead to the occurrence of various diseases, such as cancer, neurodegenerative diseases and diabetes, etc. [39, 40]. From this aspect, lysosome stress caused by hunger, unfolded protein reaction, oxidative stress and viral infection, is the primary prerequisite for its participation in autophagy [39]. This process is regulated by upstream pathways, of which transcription factor EB (TFEB) plays a particularly important role [41]. When cells are starving or mitochondria are damaged, TFEB located on the lysosome membrane can be activated for dephosphorylation in the nucleus, helping to regulate innate and adaptive immunity, cell metabolism, cell differentiation, and other important life processes. Phosphorylation of mTOR and ERK upstream of TFEB also affects expression of key proteins in the lysosomal-TFEB autophagy pathway [42, 43]. However, it is unclear whether upstream targets regulating TFEB nuclear translocation are consistent in different hepatocyte models after CDs exposure and whether the upstream targets affect lysosomal-TFEB autophagic flux. The current study addresses these questions to better define the mechanism of CDs-induced hepatotoxicity.

This study establishes mouse liver macrophage KUP5 cells and mouse normal liver parenchymal AML12 cells models in vitro and analyzes the different toxic effects and death outcomes caused by CDs exposure. To explore the mechanism of CDs activating the TFEB autophagy pathway through the lysosomal stress response to cause different hepatocyte death outcomes, and then to comprehensively evaluate the risk of hepatotoxicity of CDs exposure.

## Results

### Physicochemical properties and cytotoxicity of CDs

**Fig. 1:**
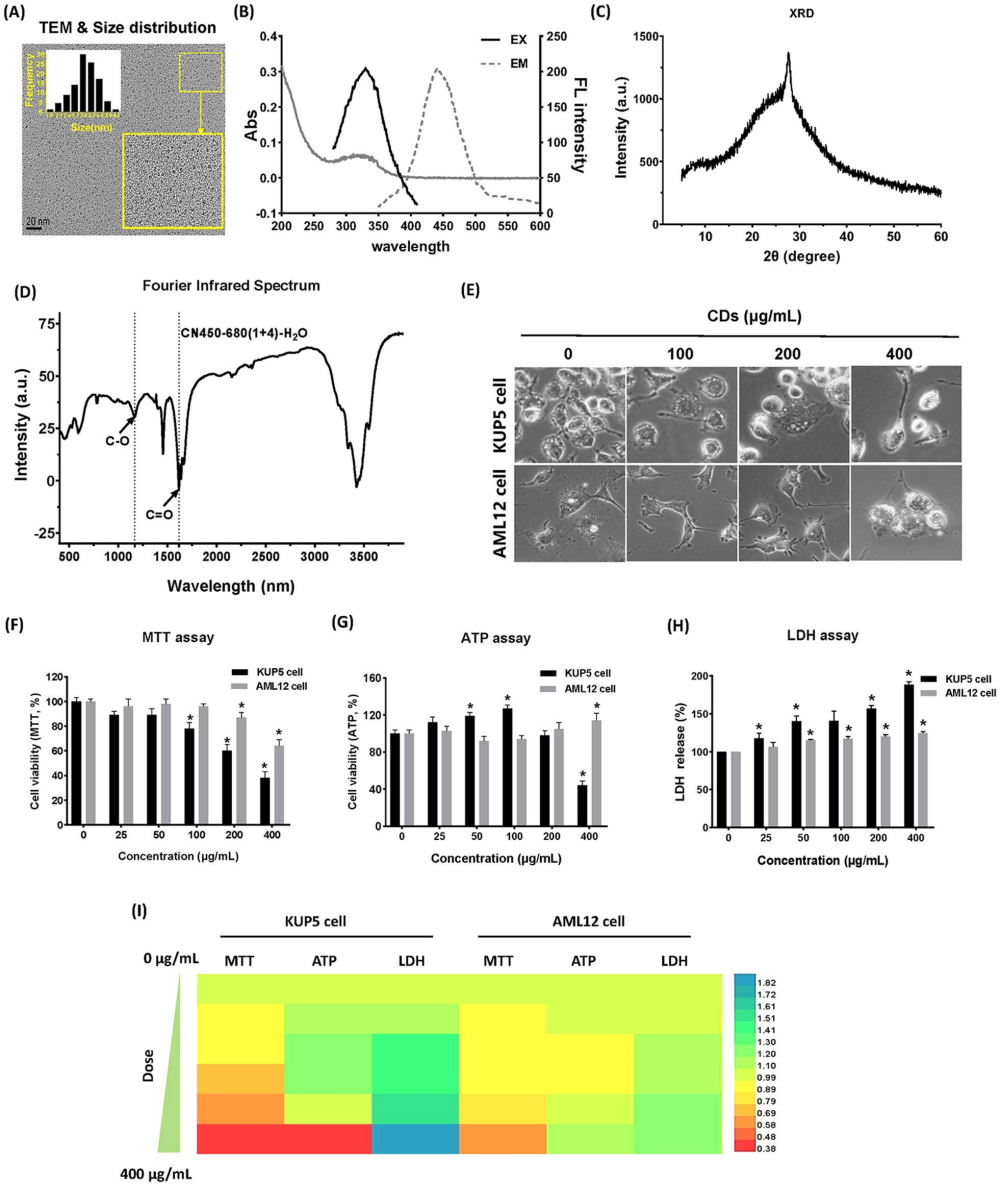
Characterization of CDs and determination of basic toxicity parameters. **A** TEM images and size distribution of CDs. Bar = 20 nm. **B** UV–Vis absorption spectra and fluorescence excitation-emission spectra of CDs. **C** and **D** X-ray Diffraction (XRD) and Fourier Transform Infrared Spectroscopy (FTIR) of CDs. **E** Morphological profiles of KUP5 and AML12 cells treated with 0, 100, 200, and 400 μg/mL CDs for 24 h. **F–H** Cell viability, ATP level, and LDH release rate of KUP5 and AML12 cells were evaluated 24 h after treatment with 0, 25, 50, 100, 200, and 400 μg/mL CDs. **I** The cell viability, ATP, and LDH levels of KUP5 and AML12 cells were visualized by heatmap. N = 3 replications. All values are mean ± SD. The statistical significance of differences was evaluated by one-way ANOVA. Compared with the control group. *, *P* < 0.05

Well characterized CDs were utilized for this study. In brief, the TEM results showed that CDs prepared in the experiment were uniformly distributed in a spherical shape without large agglomeration, and the particles exhibited a narrow size distribution at 3.0–3.3 nm (Fig. 1A). UV–Vis analysis showed that the synthesized CDs had luminescent properties and the maximum emission wavelength was around 450 nm (Fig. 1B), which laid the foundation for intracellular localization experiments. Figure 1C shows a wide base steep diffraction peak at the position of 27.5 degrees, indicating that the material has a lamellar microcrystalline structure. Combined with Fig. 1D, it can be found that oxygen-containing functional groups are functionalized on the CDs surface, such as C–O (1140 cm^−1^), C=O (1720 cm^−1^) and C–OH (1252 cm^−1^). Through the characterization results, it can be found that the synthetic carbon point is dominated by carbon element, and the surface functionalization leads to the increase of oxygen content. A set of parameters were investigated in KUP5 and AML12 cells lines to obtain toxicological profiling that represented how the two liver cell types were impacted by CDs. Prior studies have shown that the toxicological responses of phagocytes and parenchymal cells are different [15]. The first experiment assessed the morphology of the two kinds of cells before and after treatment with 0–400 μg/mL CDs. The results showed that when KUP5 cells were not treated with CDs, there were fewer pseudopodia and most of them were oval, indicating that the KUP5 cells were in an inactive state. With the increase of CDs treatment dose, the number of KUP5 cells decreased, but the cell pseudopodia branch became longer, indicating that KUP5 cells were activated in the process of uptake and response of xenobiotics. When AML12 cells were not treated with CDs, they had short branching structures, which played a role of connection and support in liver tissue. However, with the increase of CDs treatment dose, the pseudopodia of AML12 cells gradually decreased, indicating that the cells were damaged and became round (Fig. 1E). The cytotoxic potency of CDs was then conducted using three cytotoxicity assays, MTT, adenosine triphosphate (ATP) assay and lactate dehydrogenase (LDH) assay, and it was found that while the viability of both KUP5 and AML12 cells decreased with increasing CDs concentration, the decrease in KUP5 cell viability was more obvious, with these cells reduced by 50% at a 400 μg/mL CDs concentration (Fig. 1F). The MTT results were confirmed using a luminescence-based viability assay that determines ATP content and an LDH assay that assesses the level of plasma membrane damage. These experiments showed that intracellular ATP decreased and the LDH release rate increased with higher CDs concentrations. While KUP5 cells in the high-dose group showed a significant decrease in ATP content and an increase in LDH release rate (*P* < 0.05), the toxic effect of CDs on AML12 cells was lower (Fig. 1G–H). The cytotoxic profiles of the two cell lines in response to CDs exposure were compared by plotting cell-line associated cell viability (Fig. 1I). Overall, CDs had a greater cytotoxic impact on KUP5 than AML12 cells in a concentration-dependent manner.

### CDs induce cellular oxidative stress and apoptosis in KUP5 and AML12 cells

**Fig. 2:**
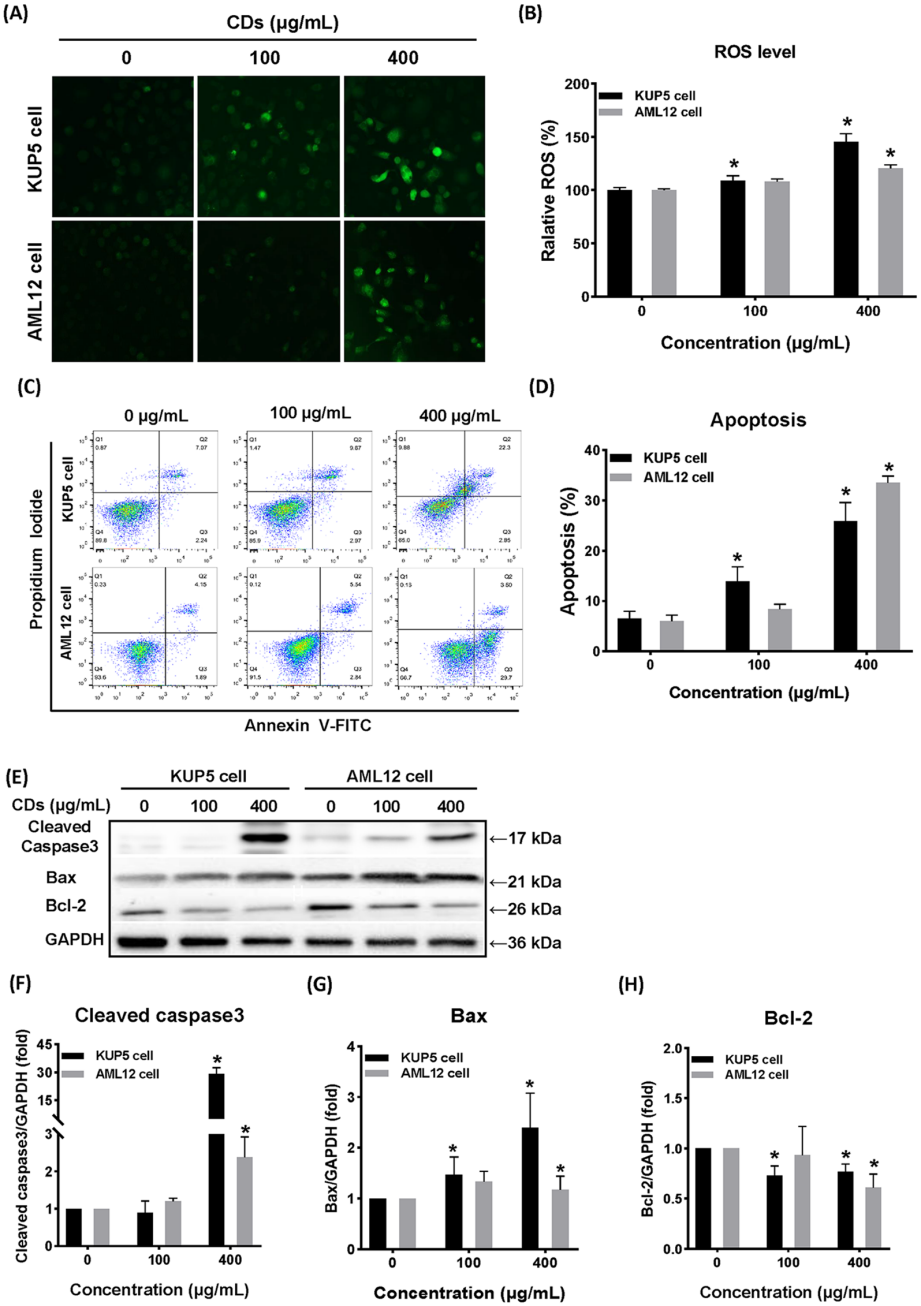
CDs induced different degrees of apoptosis and oxidative stress in two liver cell types. **A **and **B** are the distribution and quantification results of ROS in KUP5 and AML12 cells after CDs treatment at 0, 100, and 400 μg/mL for 24 h. N = 3 replications. **C** and **D** Scatterplot and quantification of KUP5 and AML12 cell apoptosis after treatment with 0, 100 and 400 μg/mL CDs for 24 h. Quantitative criterion was that the total apoptosis rate (Q1 + Q2) of the control group was lower than 10%. **E**–**H** Expression of apoptosis-related proteins Cleaved caspase3, Bax, and Bcl-2 in KUP5 and AML12 cells after CD treatment at 0, 100 and 400 μg/mL for 12 h. All values are mean ± SD. The statistical significance of differences was evaluated by one-way ANOVA. Compared with the control group. *, *P* < 0.05

Nanoparticles activate an oxidative stress response, which is mainly caused by generation of reactive oxygen species (ROS). ROS production was measured in response to CDs treatment and the results showed that in the 400 μg/mL treatment group, both cells had strong green fluorescence (Fig. 2A, B). Interestingly, the ROS level of KUP5 cells was higher than that in AML12 cells at the same exposure level of CDs (400 μg/mL). The excess ROS generation may induce an array of physiopathologic outcomes including apoptosis, inflammation, and DNA damage. To investigate the outcome and possible causes of CDs-induced injury of KUP5 and AML12 cells, the apoptosis of both cell types was measured. Figure 2C, D show changes in apoptosis of KUP5 and AML12 cells after exposure to CDs concentrations of 0,100 and 400 μg/mL. While exposure to 100 μg/mL CDs could cause apoptosis of KUP5 cells, 400 μg/mL CDs was required to induce apoptosis of AML12 cells. Further analysis showed that the key apoptosis regulator, Bcl-2, was expressed at a low level in both cell types, while the pro-apoptotic protein, Bax, gradually increased as the CDs concentration increased. The downstream caspase3 protein increased most significantly in the 400 μg/mL dose group. These results suggest that cells with a higher rate of apoptosis may be caused by greater oxidative stress, and the response of KUP5 cells to CDs-induced oxidative stress and apoptosis case was stronger than AML12 cells.

### CDs cause different autophagic flux processes in the two liver cell types

**Fig. 3:**
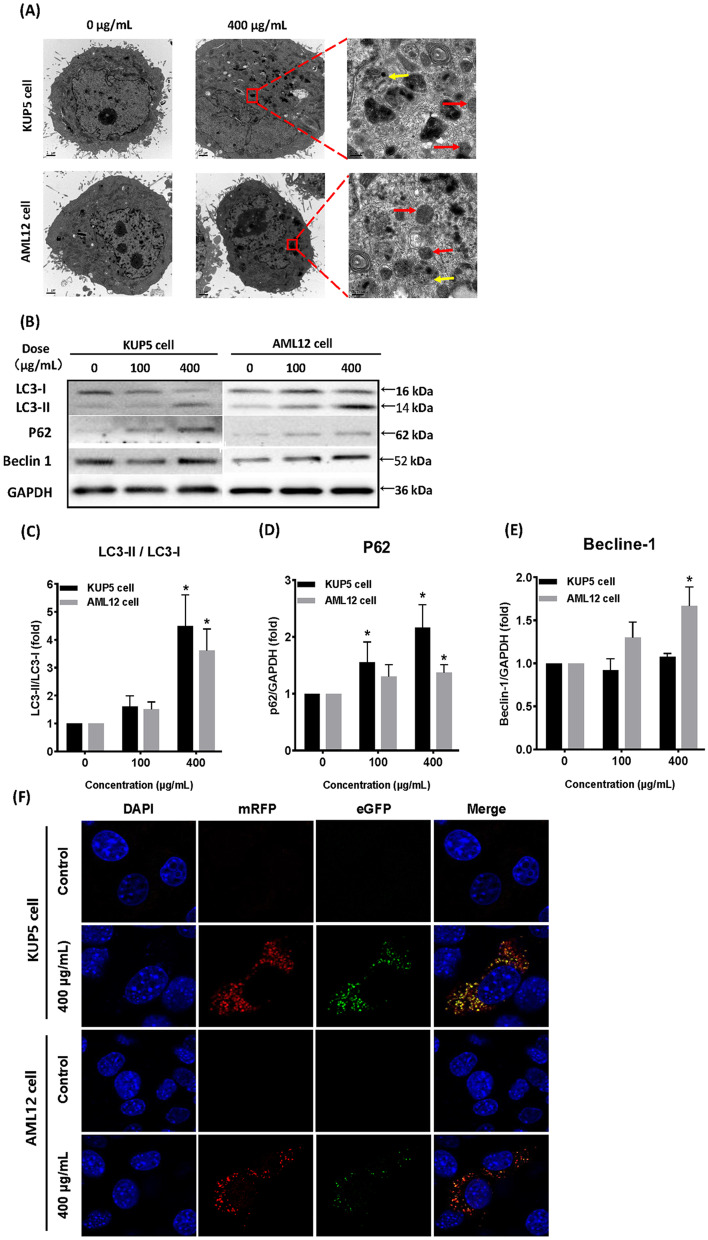
CDs caused different autophagic flux processes in the two hepatocytes. **A** TEM observations showed changes to the intracellular subcellular structure of KUP5 and AML12 cells after 12 h treatment with 0 and 400 μg/mL CDs. The red arrow shows the autophagosomes and the yellow arrow shows the autophagolysosomes. **B**–**E** Expression of autophagy-related proteins LC3-II/LC3-I, p62 and Becline1 in KUP5 and AML12 cells after 6 h treatment with 0, 100 and 400 μg/mL CDs. **F** Transfection of mRFP-GFP-LC3 plasmid into KUP5 and AML12 cells. Changes in autophagy after treatment with 0 and 400 μg/mL CDs for 12 h were observed by confocal laser microscopy. Blue is the nucleus; yellow-green fluorescence after fusion indicates that autophagic flux is blocked; red fluorescence after fusion mostly indicates that autophagic flux is unimpeded. N = 3 replications. All values are mean ± SD. The statistical significance of differences was evaluated by one-way ANOVA. Compared with the control group. *, *P* < 0.05

While apoptosis is the obvious mechanism of CDs-induced cell death for both KUP5 and AML12 cells, autophagy was also shown to play an important regulatory role in regulating the fate of these cell types. TEM results showed that after 400 μg/mL CDs treatment, both liver cells showed typical structures of autophagy: autophagy autophagosome (red arrow) and autophagolysosome (yellow arrow) (Fig. 3A). Autophagosome is a characteristic structure in the process of autophagy, that is, CDs or damaged organelles entering the cell are wrapped by small vesicles and formed a closed spherical structure. Combined with the results in Fig. 2, it was found that 400 μg/mL induced apoptosis level was also the highest, suggesting that autophagy and apoptosis occurred simultaneously in KUP5 and AML12 cells. Expression of intracellular autophagy-related proteins was verified by western blot (Fig. 3B–E). While LC3-I expression gradually decreased, LC3-II expression increased. In addition, we found that the expression levels of autophagy signature proteins p62 and Beclin1 in KUP5 cells and AML12 cells were gradually increased, suggesting that autophagy occurred in both cells. However, the expression of p62 protein in KUP5 cells increased more significantly. Considering that autophagy is a dynamic process, whether the inconsistent mortality and apoptosis degree of the two kinds of cells are related to autophagic flux, we then investigated autophagic flux of KUP5 and AML12 cells in the absence and presence of CDs by combining the distribution of red and green fluorescence after transfection of mRFP-GFP-LC3 plasmid into the cells. Theoretically, both red and green fluorescence exist in the early stages of autophagosome formation, autophagosomes will fuse with lysosomes under the premise that autophagic flux is unimpeded, and green fluorescence will be quenching in response to the acidic environment of lysosomes. Thus, observation of fluorescence imaging after merging can assess whether autophagic flux remains unobstructed. Interestingly, after treatment with 400 μg/mL CDs, KUP5 cells primarily showed yellow-green fluorescence, indicating that the autophagosome and lysosome could not fuse normally and autophagic flux was blocked, while AML12 cells showed mainly red fluorescence, indicating that the autophagosome and lysosome could fuse normally, and autophagic flux was unimpeded (Fig. 3F).

### CDs-induced autophagy mediated different apoptosis outcomes in the two hepatocyte types

**Fig. 4:**
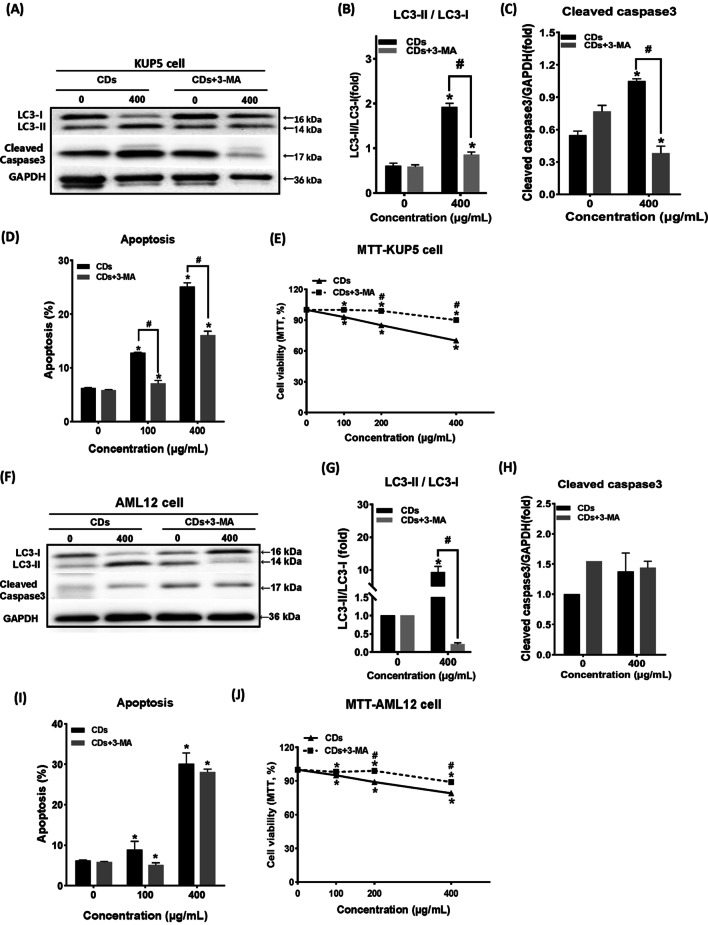
CD-induced autophagy mediated different apoptosis outcomes in the two cell types. **A**–**J** After 12 h treatment with 0 and 400 μg/mL CDs, expression of autophagy-related proteins, LC3 and Cleaved caspase3, were detected in KUP5 and AML12 cells. In the autophagy inhibitor group, KUP5 and AML12 cells were pretreated with 1 mM 3-MA for 2 h before treatment. N = 3 replications. All values are mean ± SD. The statistical significance of differences was evaluated by one-way ANOVA. Compared with the control group. *, *P* < 0.05. ^#^Compared with the untreated 3-MA group, *P* < 0.05

To further understand the role of autophagy in CDs toxicity, the autophagy inhibitor, 3-Methyladenine (3-MA), was used for subsequent verification. 3-MA inhibits autophagosome formation and can be used as an inhibitor of the first stage of autophagy. Results showed that the LC3-II/LC3-I ratio in KUP5 cells decreased after 3-MA treatment (Fig. 4A, B), suggesting that 3-MA inhibited autophagosome formation, thus preventing the accumulation of autophagosomes in KUP5 cells. Expression of active caspase3 also decreased (Fig. 4C) (*P* < 0.05) indicated that the apoptosis of KUP5 cells decreased. After 3-MA treatment, KUP5 cell activity increased and apoptosis decreased (Fig. 4D, E). These findings suggest that autophagosome accumulation caused by autophagic flux blockage in KUP5 cells may correlate with CDs-induced apoptosis. The same experiment was conducted on AML12 cells, and results showed that the ratio of LC3-II/LC3-I also decreased (Fig. 4F–J), indicating that the formation of autophagosomes was inhibited. However, caspase3 expression and level of apoptosis did not decrease after 3-MA treatment (*P* < 0.05). In view of this result, we believe that the occurrence of apoptosis of AML12 cells is not caused by the accumulation of autophagosomes, and there is no obvious correlation between the unimpeded or not of autophagic flux in AML12 cells and apoptosis. Thus, CDs may induce different apoptosis pathways in KUP5 and AML12 cells by causing different types of autophagic flux.

### CDs result in different degrees of lysosomal damage in KUP5 and AML12 cells

**Fig. 5:**
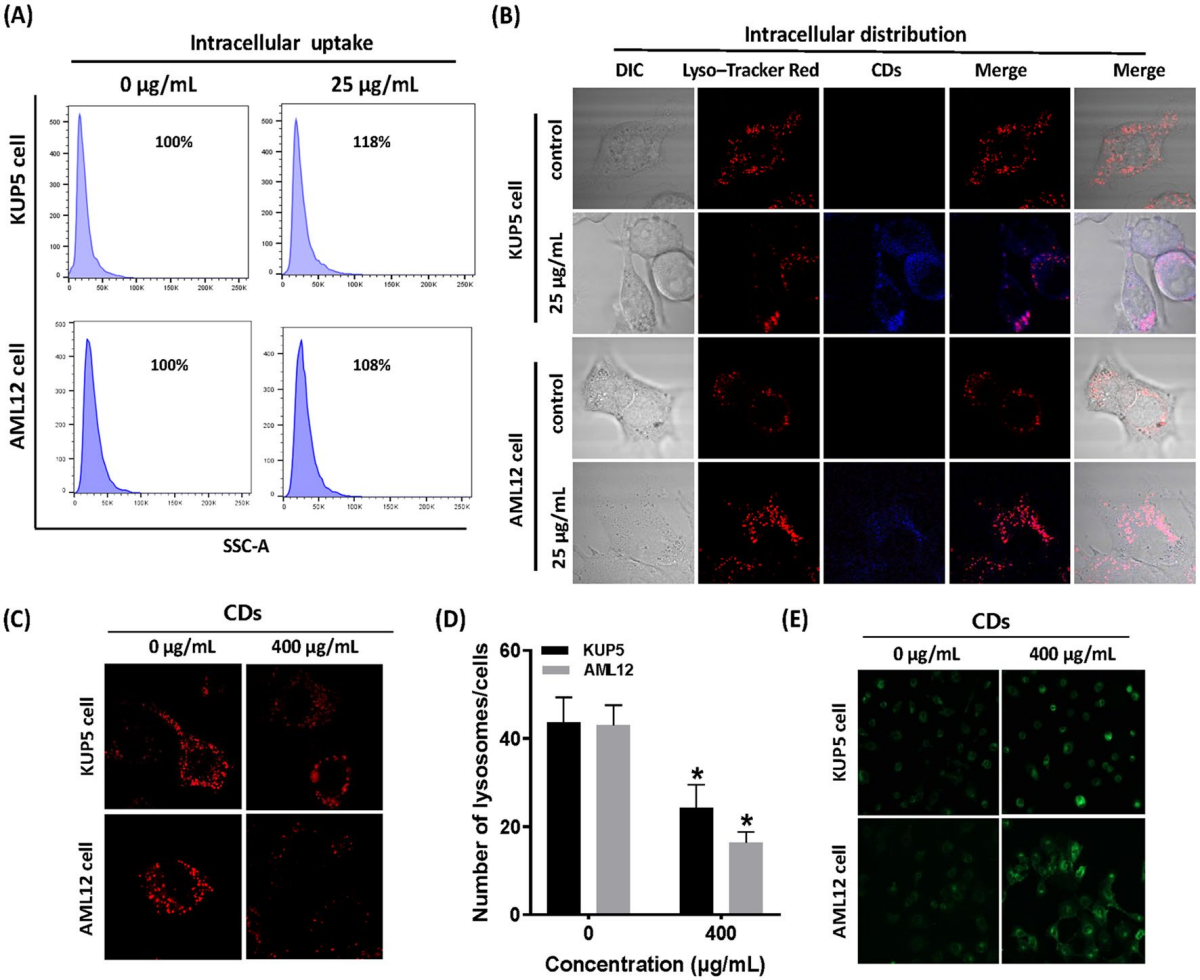
CDs resulted in different degrees of lysosomal damage in the two cell types. **A** Flow cytometry was used to analyze cell uptake of KUP5 and AML12 cells treated with 0 and 25 μg/mL CDs for 1 h. **B** Confocal microscopy was used to observe the distribution relationship between lysosomes and CDs in KUP5 and AML12 cells after 0 and 25 μg/mL CDs treatment for 1 h. LysoTracker Red is a lysosomal red fluorescent probe and blue is the distribution of CDs in cells. **C**, **D** Showed changes in the distribution and number of lysosomes labeled with LysoTracker Red in the treated and control groups. **E** Changes of lysosomal pH values in KUP5 and AML12 cells after 0 and 400 μg/mL CDs treatment for 1 h. LysoTracker Green DND is a lysosomal green fluorescent probe used to display changes in the acidic environment of the lysosome. N = 3 replications. All values are mean ± SD. The statistical significance of differences was evaluated by one-way ANOVA. Compared with the control group. *, *P* < 0.05

The intracellular uptake of nano-particles (NPs) is strongly associated with a variety of cytotoxicity-related effects, including oxidative stress, apoptosis, and autophagy [13, 44]. To explore the different mechanisms of CDs-induced toxicity of KUP5 and AML12 cells, flow cytometry was used to assess the intracellular uptake of CDs by the two cell types. We chose a dose that did not cause significant cell death (25 μg/mL) for exposure to better determine the level of uptake of cells under normal physiological conditions, the results showed that KUP5 cells uptake was slightly higher than AML12 cells uptake (Fig. 5A). The distribution of CDs uptake by the two kinds of cells was further assessed using LysoTracker Red fluorescence probe, and the results showed that both cells could uptake CDs, and the ingested CDs were mainly distributed in the lysosome (Fig. 5B). It is important to note that impairment of lysosomal function is a key event to the blockade of autophagic flux. To assess whether CDs also target lysosomes and interfere with lysosomal function and/or stability, the relationship between CDs-induced autophagic flux and lysosomal function was analyzed. A significantly lower number of lysosomes were present in CDs-treated cells and the stable pH environment in lysosomes was altered, indicating that the function and homeostasis of lysosomes in KUP5 and AML12 cells were adversely affected by CDs ingestion (Fig. 5C, D). These results drew attention to the impact of CDs on lysosomal stress regulation.

### Involvement of CDs-induced TFEB nuclear translocation in lysosomal stress regulation

**Fig. 6:**
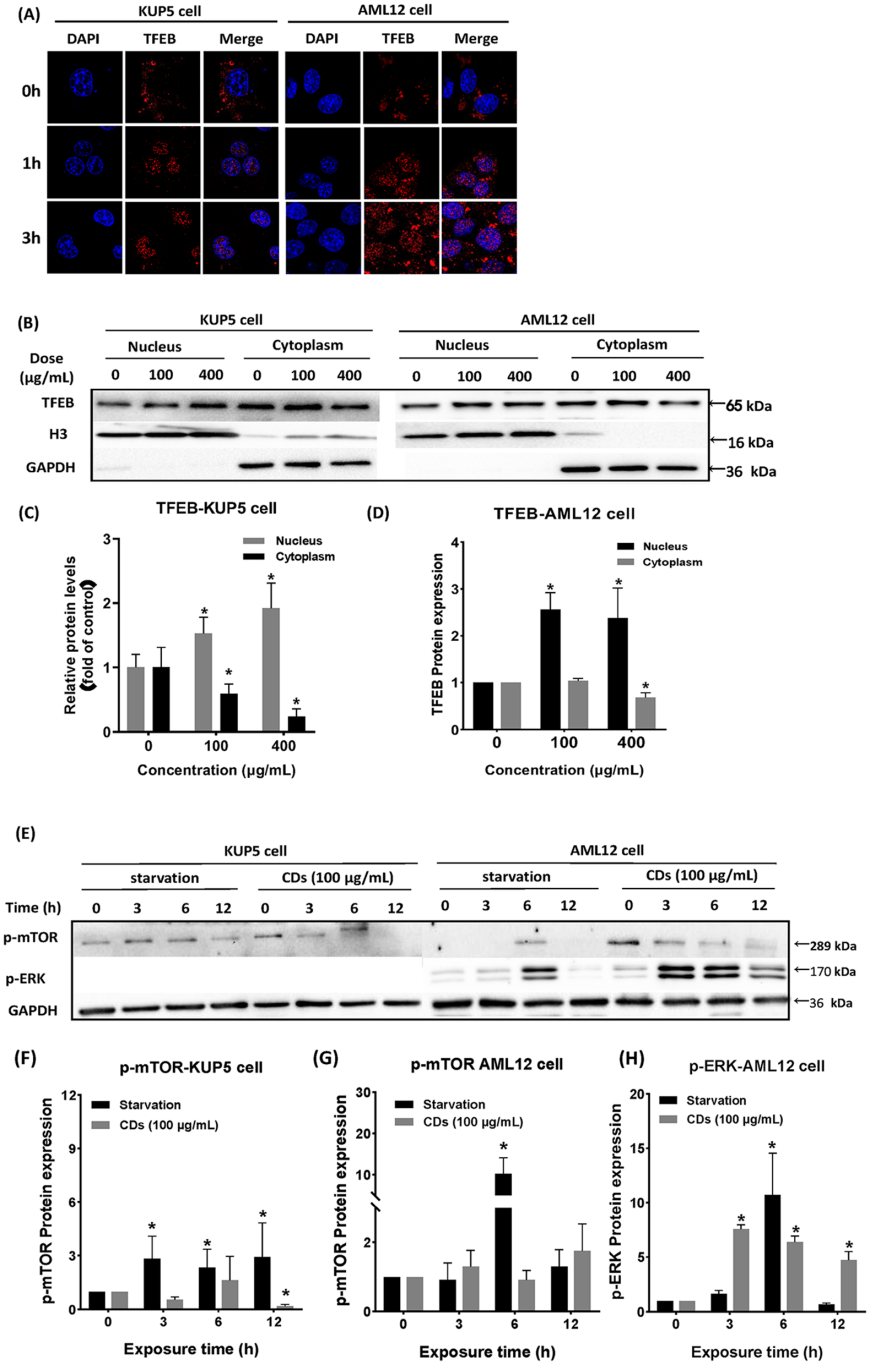
CD-induced TFEB nuclear translocation may be involved in lysosomal stress regulation. **A** Nuclear translocation of TFEB in KUP5 and AML12 cells after 0, 1, and 3 h were treated with 400 μg/mL CDs. KUP5 cells were observed by immunofluorescence and AML12 cells by mCherry-TFEB transfection. **B**–**D** TFEB expression in the nucleus and cytoplasm of KUP5 and AML12 cells treated with 400 μg/mL CDs for 3 h. **E**–**H** p-mTOR and p-ERK expression in KUP5 and AML12 cells after treatment with DMEM and 100 μg/mL CDs for 0, 3, 6, and 12 h. N = 3 replications. All values are mean ± SD. The statistical significance of differences was evaluated by one-way ANOVA. Compared with the control group. *, *P* < 0.05

Lysosomes participate in the fusion stage of autophagosomes. Based on prior studies, it was considered whether there may be a correlation between lysosomal stress and autophagy. TFEB is a master controller of lysosomal biogenesis and function and plays a major role in endo-lysosome expansion associated with lysosomal stress. Results of immunofluorescence and plasmid transfection showed that nuclear translocation of TFEB occurred in both cell types after treatment with 400 μg/mL CDs for 1 h, indicating that the lysosomal stress response was induced in the early stage of CDs exposure (Fig. 6A). Western blot results further demonstrated that after lysosomal stress, TFEB dephosphorylation entered the nucleus to participate in subsequent regulation (Fig. 6C, E). Extracellular signal-regulated kinase 2 (ERK2) and mammalian target of rapamycin complex 1 (mTORC1) are used for exogenous regulation of TFEB entry into the nucleus. To further analyze differences in the induction of TFEB into the nucleus by exogenous factors, expression of p-mTOR and p-ERK were measured in KUP5 and AML12 cells after treatment with 100 μg/mL CDs, using a starvation group as the control. Results showed that p-mTOR expression was down-regulated in KUP5 cells after 3 h of CDs treatment (Fig. 6G), and it was shown in Fig. 6A that TFEB was transferred into the nucleus after 1 h of treatment. Since nuclear translocation of TFEB is closely related to its own dephosphorylation, low expression of p-mTOR can cause TFEB dephosphorylation. It is concluded that TFEB nuclear translocation caused by 100 μg/mL CDs treatment of KUP5 cells is dependent on the phosphorylation modification of mTOR. Expression of p-ERK was not found in KUP5 cells after CDs treatment, suggesting that nuclear translocation of TFEB in KUP5 cells was not ERK-dependent. The same experiments were conducted on AML12 cells and showed that with a longer CDs treatment time, active p-mTOR and p-ERK expression decreased in a dose-dependent manner. These results indicate that the nuclear translocation of TFEB in AML12 cells is closely related to the regulation of mTOR and ERK.

**Fig. 7:**
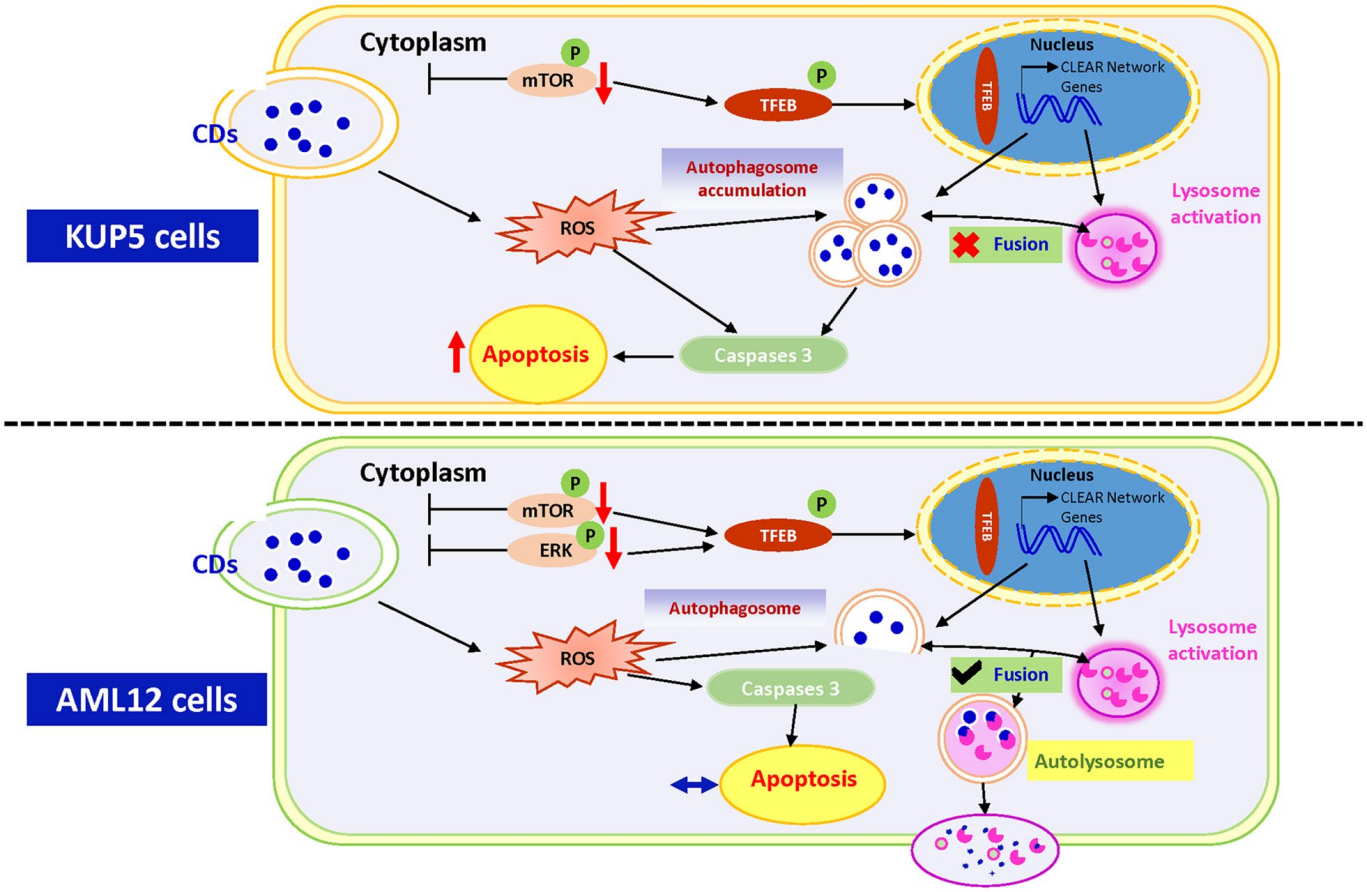
Graphical abstract. CDs leads to the production of ROS in both liver cells and autophagy activation by activating the TFEB-lysosome pathway. Of note, CDs can induce the accumulation of autophagosomes in KUP5 cells, leading to the arrest of autophagic flux and ultimately promoting cell apoptosis. Autophagic flux was unobstructed in AML12 cells, and the apoptosis level was not related to the progress of autophagic flux

Figure 7 summarizes how CDs can cause KUP5 and AML12 cells to undergo different levels of apoptosis by inducing distinct autophagy mechanisms. While KUP5 cells blocked autophagic flux, AML12 cells had unimpeded autophagic flux. These differences may be due to discrepancies in upstream pathways that mediate TFEB nuclear translocation, with autophagosome accumulation inducing a more significant level of apoptosis in KUP5 cells.

## Discussion

In the present study, CDs were shown to have a significantly different toxic effect on KUP5 and AML12 cells. CDs accelerated autophagosome accumulation by blocking autophagic flux of immune-derived KUP5 cells, and ultimately increased apoptosis. However, non-immune-derived AML12 cells displayed strong self-tolerance, with unimpeded autophagic flux and the usage of the autophagosome formation inhibitor, 3-MA, had no significant effect on AML12 cells apoptosis. Previous studies have shown that CDs can cause both lysosome reduction and lysosomal stress and are found to be co-localized with lysosomes in two kinds of liver cells [21]. Thus, this study assessed the expression of TFEB, a nuclear transcription factor affected by lysosomal stress during autophagy. Nuclear translocation of TFEB is regulated by phosphorylation of mTOR and ERK so experiments were conducted to assess whether the different performance of the two hepatocytes during autophagy may be caused by discrepancies in the upstream regulatory network of TFEB. Results indicated that the activation of TFEB-lysosome pathway in normal liver parenchyma cells requires dual regulation of upstream ERK and mTOR, but TFEB activation caused by lysosomal stress in KUP5 cells is only regulated by mTOR. Among the two cells, KUP5 cells had more sensitive immune activation properties. Figure 7 provides a further overview of the mechanism.

CDs are a new one-dimensional carbon material with good biocompatibility [45–48]. However, prior studies have found that CDs application may have negative outcomes [21, 49, 50], especially given the potential damage they cause to the liver. In this study, KUP5 and AML12 cells models were established in vitro, to analyze the response of different cell types to the same toxin from the perspective of liver injury, a topic not widely covered by prior studies. Results showed that CDs could reduce the cell viability of both KUP5 and AML12 cells but each cell responded in a distinct way. When both cell types were exposed to the same CDs dose, KUP5 cells exhibited more severe energy metabolic disorders and membrane damage, a finding consistent with that shown by Pang et al. [15]. KUP5 cell exposure to low CDs doses significantly reduced cell vitality, which may be because these cells are macrophages, participating in the immune response and responsible for taking up and presenting foreign particles. This may make KUP5 cells more sensitive to CDs exposure. It is also possible that the two kinds of cells activate different death pathways. What’s more, AML12 cells may have adaptive immunomodulatory mechanisms that improve the response to CDs, leading to more apoptosis and cell viability than KUP5 cells in response to higher CDs doses. Here, adaptive immune regulation can be understood as AML12 cells may clear damaged cells by increasing apoptosis so that this cell type can maintain a certain level of cellular viability in an adverse environment [51, 52]. Both liver cell types also showed high expression of the pro-apoptotic protein, Bax, and Cleaved caspase3, and low expression of the anti-apoptotic protein, Bcl-2. ROS induced by nanomaterials is the main mechanism of oxidative stress [53, 54], and this process correlates with apoptosis to a certain extent [14, 55]. Therefore, ROS levels in the two cells were measured as a proxy for apoptosis. Results showed that both KUP5 and AML12 cells stimulated strong oxidative stress under high-dose exposure. Thus, CDs may cause apoptosis of KUP5 and AML12 cells by activating two kinds of intracellular oxidative stress, and the adaptive immune regulation of AML12 cells may enable them to have higher cellular viability under the same level of exposure.

In addition to measuring apoptosis, the level of autophagy and related sub-organelles was assessed in the two liver cells. Lysosomes, organelles involved in autophagy, can influence cell fate [56]. TEM results clearly showed the existence of autophagosomes in both KUP5 and AML12 cells, indicating that both apoptosis and autophagy occurred in the two liver cell types. Western blot results also showed that autophagy signature proteins, the LC3-II/LC3-I ratio and Beclin1, were increased in KUP5 and AML12 cells in response to CDs. Beclin1 can regulate the formation of autophagy precursors and guide the aggregation of related proteins in the autophagosome membrane. The above results indicate that autophagic flux occurred in both liver cell types. Besides, p62 protein expression was significantly higher in KUP5 than AML12 cells. p62 is an ubiquitin-binding protein that participates in autophagosome degradation during autophagy [57, 58]. However, p62 expression in AML12 cells did not show a significant increase, this indicates that autophagic flux in AML12 cells is unimpeded. Autophagic flux is usually divided into the formation of autophagosomes, autophagosome fusion with lysosomes that forms the autophagy-lysosome, and degradation of the autophagy-lysosome [40]. Results from this study showed that p62 in KUP5 cells did not participate in autophagosome degradation and allowed them to accumulate in large numbers, however, autophagic flux was unimpeded in AML12 cells. It was speculated that there might be an autophagic flux block in KUP5 cells, autophagosomes fail to fuse with lysosomes and leading to autophagosome accumulation. To verify this hypothesis, the fluorescence distribution of mRFP-GFP-LC3 plasmids was assessed after transfection by laser confocal microscopy. Both red and green fluorescence was expressed in normal cells, and when the autophagosome and lysosome fuse smoothly, the green fluorescence will be quenched due to the influence of acidic environment in the lysosome and the final fluorescence after the merge was red. Fluorescence images also showed the autophagosome failed to fuse with the lysosome, and a large accumulation of autophagosomes in KUP5 cells, resulting in yellow-green fluorescence. CDs blocked the autophagic flux in KUP5 cells, an important distinction from the autophagy outcome of AML12 cells.

Prior studies have shown that different degrees of apoptosis and autophagy exist in KUP5 and AML12 cells, and there are differences in the way each cell type responds to CDs exposure. Whether there is a mutual influence between these two death modes is a subject for further study. When an inhibitor of autophagosome formation, 3-MA, was applied to KUP5 and AML12 cells, the LC3-II/LC3-I ratio decreased in both liver cell types, indicating that autophagosome formation was successfully inhibited [59]. Cleaved caspase3 expression decreased significantly in KUP5 cells but not significant changes in AML12 cells, suggesting that 3-MA prevents autophagosome accumulation and decreases apoptosis in KUP5 cells. However, since there was no accumulation of autophagosomes in AML12 cells, 3-MA could not affect apoptosis by alleviating autophagic flux arrest, which was different from KUP5 cells. In conclusion, apoptosis of KUP5 cells may be caused by autophagosome accumulation, which has been confirmed by existing studies [60, 61], however AML12 cell apoptosis may involve other pathways [62].

Results from this study show that two liver cell types can lead to different cell death outcomes through distinct autophagic flux processes. Since lysosomes can serve as the core of autophagosome degradation during autophagy [63], the basic uptake of KUP5 and AML12 cells and the extent of lysosome damage were assessed after exposure to CDs. Results showed that uptake of KUP5 cells was slightly higher than that of AML12 cells, which may be because KUP5 cells are macrophages and have higher uptake capacity. Interestingly, CDs ingested by the two types of cells were mainly distributed in lysosomes. Thus, the accumulation of CDs in lysosomes may be closely related to CDs-induced autophagy, in which case the distribution of CDs in lysosomes would cause lysosomal damage. Findings showed that CDs reduced the number of lysosomes in both liver cell types, changed the acidic microenvironment of the lysosomes, and reduced the pH value. In summary, CDs treatment resulted in lysosomal stress in both liver cell types.

TFEB is an important nutritional and stress sensing nuclear transcription factor [41, 42] which can regulate lysosome biogenesis. Under normal conditions, TFEB is phosphorylated and localized in the cytoplasm, but when cells are starved or experiencing lysosomal stress, TFEB is dephosphorylated and enters the nucleus [43]. Nuclear translocation of TFEB in KUP5 and AML12 cells after CDs treatment for different times were determined by immunofluorescence and plasmid transfection methods, respectively. Results showed dephosphorylation and nuclear translocation of TFEB in the early stage of CDs treatment (1 h), suggesting that TFEB activation occurred in both types of hepatocytes. The upstream pathway of TFEB activation was also determined in our study. TFEB is regulated by phosphorylation of mTOR [41] and when mTOR is inhibited, TFEB that isn’t phosphorylated can be dephosphorylated into the nucleus and participate in subsequent lysosomal stress response and autophagy. In addition, ERK2 activation mediates the dephosphorylation of TFEB [41, 64]. For this purpose, expression of p-mTOR and p-ERK in the two liver cell types was determined in the starvation and control groups. Results suggested that nuclear translocation regulation of TFEB in KUP5 cells was only mTOR dependent since inhibition of mTOR phosphorylation was observed after 3 h of CDs exposure while TFEB was dephosphorylated and translocated to the nucleus. Unfortunately, ERK expression was not detected in KUP5 cells. The difference is that nuclear translocation of TFEB can be activated by mTOR and ERK pathways in AML12 cells. This difference may be due to the fundamental difference in the activation pathway induced by CDs between the two hepatocytes. It is known that receptor tyrosine kinases, G-protein-coupled receptors and some cytokine receptors can activate ERK signal transduction pathway [65]. Different cells can activate different reaction pathways under the stimulation of xenobiotics, which may lead to the differential expression of p-ERK. In addition, some studies have found that the silencing of Kinesin family Member 2C (KIF2C) [66] and Ring Finger Protein 2 (RNF2) [67] genes can down-regulate p-ERK expression, but the specific mechanism is not clear. This also indicates that the activation of ERK pathway is the result of the mutual regulation of multiple genes, and the regulation of the expression of key genes can affect the activation of downstream p-ERK, but the specific mechanism needs to be further clarified. In this study, the regulation process of ERK signal transduction pathway induced by CDs in KUP5 cells may be different from that in AML12 cells, which may also be one of the reasons for the difference in the two liver cell death mechanisms.

## Conclusion

In summary, these data strongly suggest that CDs with good biocompatibility are unsafe for biomedical applications. CDs had a dose-dependent toxic effect on different types of normal liver cells, among them, KUP5 cells are more sensitive in the process of CDs uptake and response because of their innate advantages as immunogenic cells. KUP5 cell apoptosis was closely related to autophagosome accumulation caused by inhibition of autophagic flux. Interestingly, no autophagic flux blocking was found in AML12 cells. Further analysis showed that the nuclear translocation of TFEB in KUP5 cells was dependent on the regulation of mTOR, and in AML12 cells was dependent on the dual regulation of mTOR and ERK. Differences in the regulatory mechanisms of TFEB pathways may be responsible for differences in injury caused to the two liver cell types. This study shows the toxicity of CDs on normal liver cells at an in vitro level, and explains the difference in CDs exposure risk to different target cells. While CDs have good fluorescence characteristics and can be used for targeted imaging, the damage caused by CDs to normal cells at the same exposure level cannot be ignored. The advantage of nanomaterials in accelerating the progress of autophagic flux and assisting in the elimination of pathogens should be fully leveraged. Importantly, CDs toxicity should be fully assessed for toxicological safety before expanding their clinical use.

## Materials and methods

### Material synthesis and characterization

The CDs were prepared in two steps. Synthesis of bulk graphite carbon nitride(g-C_3_N_4_) precursor by dicyandiamide (DCDA) heat shrink method and the preparation of n-doped carbon quantum dots. Synthetic CDs were dry-roasted for 2 h on an electric heating plate at 200 °C to remove potentially doped endotoxins. Specific methods are described in a study by Yang et al. [68].

CDs were characterized by transmission electron microscopy (TEM) (Jem-2100, Japan Electronics Corporation). The synthesized CDs were diluted and dripped onto a copper wire, dried by nitrogen, observed by TEM, and measured using Nano Measure 1.2 software. Hydration particle size and Zeta potential of CDs dissolved in complete medium were measured using a Malvern laser particle size analyzer (Zetasizer Nano-ZS90, Malvern Instruments LTD, UK). UV–Vis spectra, photoluminescent (PL) spectra and fourier transform infrared spectra were determined by UV–Vis spectra (Cary 100, Agilent Technology, Singapore), F4600 fluorescence spectrometer (5J2-004, Tokyo, Japan) and fourier transform infrared spectrometer (MPA, Brook Spectroscopic Instrument Company, UK). CDs dissolved in ultra-pure water is pre-frozen for 4 h at − 80 °C and then lyophilized. The obtained solid material is used for XRD determination (Combined multifunctional horizontal X-ray Diffractometer (Ultima IV, Science, Japan)).

### Cell culture and treatment with CDs

Mouse liver macrophages (KUP5 cells) and normal mouse liver cells (AML12 cells) were purchased from RIKEN Cell Bank (Japan) and the Shanghai Institute of Cell Science, Chinese Academy of Sciences, respectively. Both cell types were cultured in Dulbecco's Modified Eagle Medium (DMEM) (Gibco, USA) with 10% fetal bovine serum (FBS) (Gibco, USA) and 1% Penicillin–Streptomycin (Hyclone, USA). KUP5 cells were also supplemented with 10 μg/mL bovine insulin (Sigma, USA) and 250 μM 1-thioglycerol (Sigma, USA). Follow-up experiments were performed when the cells reached the logarithmic growth stage. The autophagy inhibitor used in this study was 3-methyladenine (3-MA, 1 mM for 2 h).

### Cytotoxicity assessment (MTT and LDH analysis)

Cytotoxicity was measured using MTT and LDH assays. KUP5 and AML12 cells with stable growth status were cultured in 96-well plates and provided with three repeat holes. The cells were cultured until they had adhered to the walls, the upper medium was discarded, and the cells were exposed to CDs at a concentration of 0, 25, 50, 100, 200, and 400 μg/mL for 24 h. Absorbance was determined using the LDH analysis kit instructions (Beyotime Biotechnology, Shanghai, China) and the MTT analysis strategy (Sigma-Aldrich, USA).

In brief, the 96-well plate was centrifuged at 400 g for 5 min, the supernatant was co-incubated with LDH working fluids in the dark at 37 °C for 30 min, the absorbance was measured at 490 nm, and the LDH release rate was analyzed. The MTT method is primarily based on dissolution of purple methylzan by living cells using dimethyl sulfoxide (DMSO). Cell viability is positively correlated with the absorbance value. In brief, 100 μL MTT solution (volume fraction 10%) was added to each well, plates were incubated in the dark for 4 h, and 150 μL DMSO was used to dissolve the crystals. The absorbance value of the solution was detected at 490 nm using a microplate reader.

### ATP analysis

KUP5 and AML12 cells were cultured in 24-well plates at 5 × 10^4^ cells per well with three repeat holes, and exposed to CD concentrations of 0, 25, 50, 100, 200 and 400 μg/mL for 24 h. Lysis buffer (50 μL) was added to each well, the supernatant was removed after centrifugation for 5 min at 12,000 g, and ATP was measured using the instructions from the ATP Assay Kit (Beyotime Institute of Biotechnology, Shanghai, China). In brief, ATP standard solution diluted to 0.01, 0.03, 0.1, 0.3, 1, 3, and 10 μM was used to draw the standard curve and the detection working solution was prepared. The detection working solution (100 μL) and 20 μL of sample were added to each well. Relative light unit (RLU) values were determined using a luminometer and the ATP concentration was calculated according to the standard curve.

### Flow cytometry analysis

Cell apoptosis and uptake of CDs were quantified by flow cytometry (FACS Aria II, Becton Dickinson, San Jose, CA). After 12 h of CD treatment (0, 100 and 400 μg/mL), the upper culture medium was discarded, and uninternalized particles were cleaned three times with PBS. CDs uptake by KUP5 and AML12 cells was analyzed by flow cytometry. The strength of forward scatter (FSC) reflects the size of cells, and side scatter (SSC) is directly proportional to the amount of particle uptake by cells.

Annexin V/PI Apoptosis Kit (BD Biosciences, USA) was used to measure apoptosis. The cells were digested with trypsin without EDTA and collected for later use and 500 μL binding buffer was added to each tube to resuscitate the cells. Annexin V-FITC (5 μL) and PI (10 μL) were added successively, and the cells were incubated at room temperature to avoid light for 15 min. The dye-labeled cells were immediately sampled by flow cytometry and the relevant fluorescence signal was collected (Ex = 488 nm, Em = 530 nm).

### Western blot analysis

KUP5 and AML12 cells used for protein extraction were cultured in 6-well plates and treated with CDs concentrations of 0, 100 and 400 μg/mL. The upper medium was discarded and the cells were lysed as described previously [44]. The protein content was analyzed using the bicinchoninic acid (BCA) method and equal amounts (30 μg) of protein sample were loaded onto a 12.5% SDS-PAGE gel for separation, transferred to a PVDF membrane, and sealed with 5% skim milk powder diluted with TBST for 1 h. The samples were then incubated with primary antibody (1:1000) for Cleaved-caspase3, p62, Beclin1 (Cell Signal Technology (CST), USA), Bax, Bcl-2, GAPDH, TFEB, H3, p-mTOR, or p-ERK (ABclonal, Wuhan, China) at 4 °C overnight. Thermo32106ECL luminescence (Thermo Fisher Scientific, USA) measurements were performed after co-incubation with the corresponding secondary antibody (*V: V* = 1:10,000) at room temperature for 1 h. The Tanon MP imaging System (Tanon 5200, Shanghai, China) and ImageJ 1.53a were used for image acquisition and strip gray scale quantification.

### ROS assay

KUP5 and AML12 cells were cultured in 6-well plates until cell adhesion had occurred and exposed to CDs concentrations of 0, 100, and 400 μg/mL. After 24 h of exposure, both cell types were incubated with a serum-free DCFH-DA probe (Beyotime Institute of Biotechnology, Shanghai, China) (KUP5 cells 1:2000; AML12 cells 1:1000) for 30 min (away from light, 37 °C). The cells were cleaned three times with PBS and 0.5 mL PBS was reserved to cover the bottom of the dish. The green fluorescence intensity was observed with a fluorescence microscope (Ex = 488 nm) (vert. A1, ZEISS, Germany).

### Plasmid transfection

KUP5 and AML12 cells were plated on a confocal dish and cultured until the cells adhered to the wall. Lipofectamine2000 and DMEM medium without serum and double antibodies were mixed at specific ratios (KUP5 cells *V: V* = 1:500, AML12 cells *V: V* = 1:100). Plasmid (1 μg) was added to 100 μL of medium without serum and double antibodies to obtain a plasmid mixture. Lipofectamine2000 was mixed with mRFP-GFP-LC3 plasmid mixture or mCherry-TFEB plasmid mixture at 1:1 (*V:V*) and incubated at room temperature for 5 min to obtain two transfer dyes for later use. Follow-up treatment was carried out as follows: each well was treated with 300 μL dye solution for 6 h, complete culture medium for 24 h, and 400 μg/mL CDs for 1 h, 3 h, and 12 h, and the negative control was set-up. All steps were carried out at 5% CO_2_ and 37 °C. Finally, the wells were fixed with 4% paraformaldehyde and photographed with a confocal microscope (FV1000, OLYMPUS, Japan) for red, green and blue fluorescence.

### Immunofluorescence of TFEB entry into KUP5 cells

KUP5 cells at the logarithmic growth stage were plated on confocal dishes, cultured until cell adherence (5% CO_2_, 37 °C), and treated with 400 μg/mL CDs for 1 h and 3 h. The negative control group was set-up at the same time. After exposure, KUP5 cells were fixed with 4% paraformaldehyde at 37 °C for 10 min. The cells were permeated with 0.5% Triton-X100 (PBS dissolved) for 10 min at room temperature and finally sealed with 5% BSA for 30 min. After pretreatment, the cells were incubated with TFEB primary antibody at 4 °C overnight (*V:V* = 1:1000), and the corresponding fluorescent secondary antibody (*V:V* = 1:10,000) at room temperature for 1 h. DAPI was added and the cells were incubated at room temperature to avoid light for 10 min. At the end of each incubation, the cells were washed twice with TBST/PBS for 5 min. The bottom of the dish was covered with 300 μL PBS and observed with red and blue fluorescence using a confocal microscope.

### Transmission electron microscope visualization of autophagosomes

KUP5 and AML12 cells were cultured in 12-well plates and collected after exposure to CDs concentrations of 0 and 400 μg/mL for 12 h. The cells were centrifuged, treated with 0.5 mL of 2.5% glutaraldehyde at 4 °C overnight before adding 0.5 mL of 1% osmium tetroxide at 4 °C for 30 min to fix the cells. Specimens were prepared for electron microscopy according to the sequence of dehydration, immersion, embedding, clotting, sectioning and staining, and sub-organellar changes were observed under 80 kV voltage.

### Subcellular localization and PH changes of lysosomes

KUP5 and AML12 cells were inoculated on confocal dishes and infected using the same process described in Sect. “Immunofluorescence of TFEB entry into KUP5 cells”. After CDs treatment, the supernatant was discarded and incubated with LysoTracker^®^ Red DND and LysoTracker^®^ Green DND at room temperature for 10 min and 15 min, respectively (Beyotime Technology, Shanghai, China). The red and green fluorescence signals were observed by confocal microscopy after fixation with 4% paraformaldehyde at 37 °C for 10 min.

### Statistical analysis

Mean and standard deviation (SD) of multiple measurements in the experiment were calculated, and the final result was expressed as the mean ± SD. One-way ANOVA was used to compare multiple groups, and the student’s t-test was used for comparison between two samples. The difference was statistically significant based on a *P* value less than 0.05.

## Data Availability

All data and materials are included in the manuscript and figures.

## Abbreviations

KUP5 cells or KCs: Kupffer cells
AML12 cells: Parenchymal hepatic cells
CDs: Carbon dots
g-C3N4: Graphitic carbon nitrides
MPS: Mononuclear phagocyte system
ROS: Reactive oxygen species
TFEB: Transcription factor EB
ERK: Extracellular signal-regulated kinase 2
mTORC1: Mammalian target of rapamycin complex 1
KIF2C: Kinesin family Member 2C
RNF2: Ring Finger Protein 2
DMEM: Dulbecco's Modified Eagle Medium
EDTA: Ethylene diamine tetraacetic acid
FBS: Fetal bovine serum
DMSO: Dimethyl sulfoxide
ATP: Adenosine triphosphate
MTT: Methyl thiazolyl blue tetrazolium bromide
LDH: Lactate dehydrogenase
3-MA: 3-Methyladenine
NPs: Nano-particles
TEM: Transmission electron microscope
PL: Photoluminescent
XRD: X-ray diffractometer
RLU: Relative light unit
FSC: Forward scatter
SSC: Side scatter
Ex: Excitation wavelength
Em: Emission wavelength
PVDF: Polyvinylidene fluoride
